# Construction of Candida albicans Strains with ATP-Analog-Sensitive Protein Kinase A and Hog1

**DOI:** 10.1128/msphere.00095-23

**Published:** 2023-04-11

**Authors:** Zhongle Liu, Jessie MacAlpine, Nicole Robbins, Leah E. Cowen

**Affiliations:** a Department of Molecular Genetics, University of Toronto, Toronto, Ontario, Canada; University of Georgia

**Keywords:** 1NM-PP1, ATP, *Candida albicans*, Hog1, protein kinase A, genetic resource

## Abstract

Candida albicans is an opportunistic human fungal pathogen and a member of the mucosal microbiota. To survive in the host and cause disease, C. albicans utilizes several virulence traits, including the ability to respond and adapt to diverse stressors, as well as the morphogenetic switch between yeast and filamentous morphologies. While complex cellular circuitry governs these virulence attributes, the following two kinase-mediated signaling pathways play particularly critical roles in controlling these processes: the Hog1 mitogen-activated protein kinase (MAPK) cascade and the protein kinase A (PKA) pathway. Here, we describe the construction of C. albicans strains harboring substitutions in the ATP-binding pockets of Hog1 and the catalytic subunits of PKA, Tpk1, and Tpk2 to render their activities sensitive to the addition of bulky ATP analogs. Specifically, inhibition by the ATP analog 1NM-PP1 resulted in phenotypes characteristic of the corresponding homozygous deletion mutants for each kinase gene. These strains represent a toolset for the rapid and specific inhibition of PKA and Hog1 kinase activity to further understand their roles in regulating C. albicans morphogenesis and stress responses.

**IMPORTANCE** As an opportunistic pathogen in humans, the fungus Candida albicans relies on virulence traits to cause disease. They include the ability to transition from yeast to filamentous morphologies and the ability to grow in diverse environmental stress conditions, including nutrient limitation, as well as osmotic and heat shock. Previous work identified the following two kinases that play a critical role in regulating these responses: Hog1 and PKA. Here, we generated versions of each kinase that are sensitive to inhibition by a bulky ATP analog, 1NM-PP1. In the presence of the analog, kinase activity is inhibited rapidly and specifically, facilitating the analysis of both kinases in regulating C. albicans morphogenesis and stress responses. Together, these strains represent an important toolset to further our understanding of C. albicans biology and virulence.

## OBSERVATION

In humans, fungal pathogens are responsible for over 1 billion infections and 1.5 million deaths annually (1). The majority of infections in humans are caused by Candida albicans, an opportunistic pathogen that is a commensal organism in approximately half of the global population (2). C. albicans pathogenesis relies on several key virulence strategies that contribute to disease and immunopathology in human hosts, including the ability to transition from yeast to filamentous morphologies, such as hyphae (3). A core signaling pathway responsible for regulating the yeast-to-filament transition in C. albicans is the cyclic-AMP (cAMP)-protein kinase A (PKA) pathway (3). Specifically, activation of the adenylyl cyclase Cyr1 to produce cAMP relieves the PKA catalytic subunits Tpk1 and Tpk2 from inhibition by the regulatory subunit Bcy1. PKA signals are transduced to multiple transcription factors to drive filamentation. Another kinase important for C. albicans virulence is the mitogen-activated protein kinase (MAPK) Hog1, which mediates responses to diverse stressors (4–7). Hog1 also regulates hyphal morphogenesis, as well as the interaction of C. albicans with host phagocytes (8, 9). Homozygous deletion and conditional expression mutants have been used to study loss of PKA or Hog1 function in C. albicans. However, genetic methods now exist to allow for rapid and specific inhibition of protein kinases by ATP analogs (10, 11). This strategy involves mutating a gatekeeper residue in a kinase to render the ATP-binding pocket accessible to bulky ATP-analogs, such as C3-1’-naphthyl-methyl PP1 (1NM-PP1). Binding of this analog competes with the binding of ATP and renders the kinase inactive. While this approach has been used to study the function of particular kinases in C. albicans, including protein kinase C (Pkc1) (12), Cdc28 (13, 14), Ssn3 (15), and Yak1 (16), it has not been used to study the function of PKA or Hog1.

**FIG 1:**
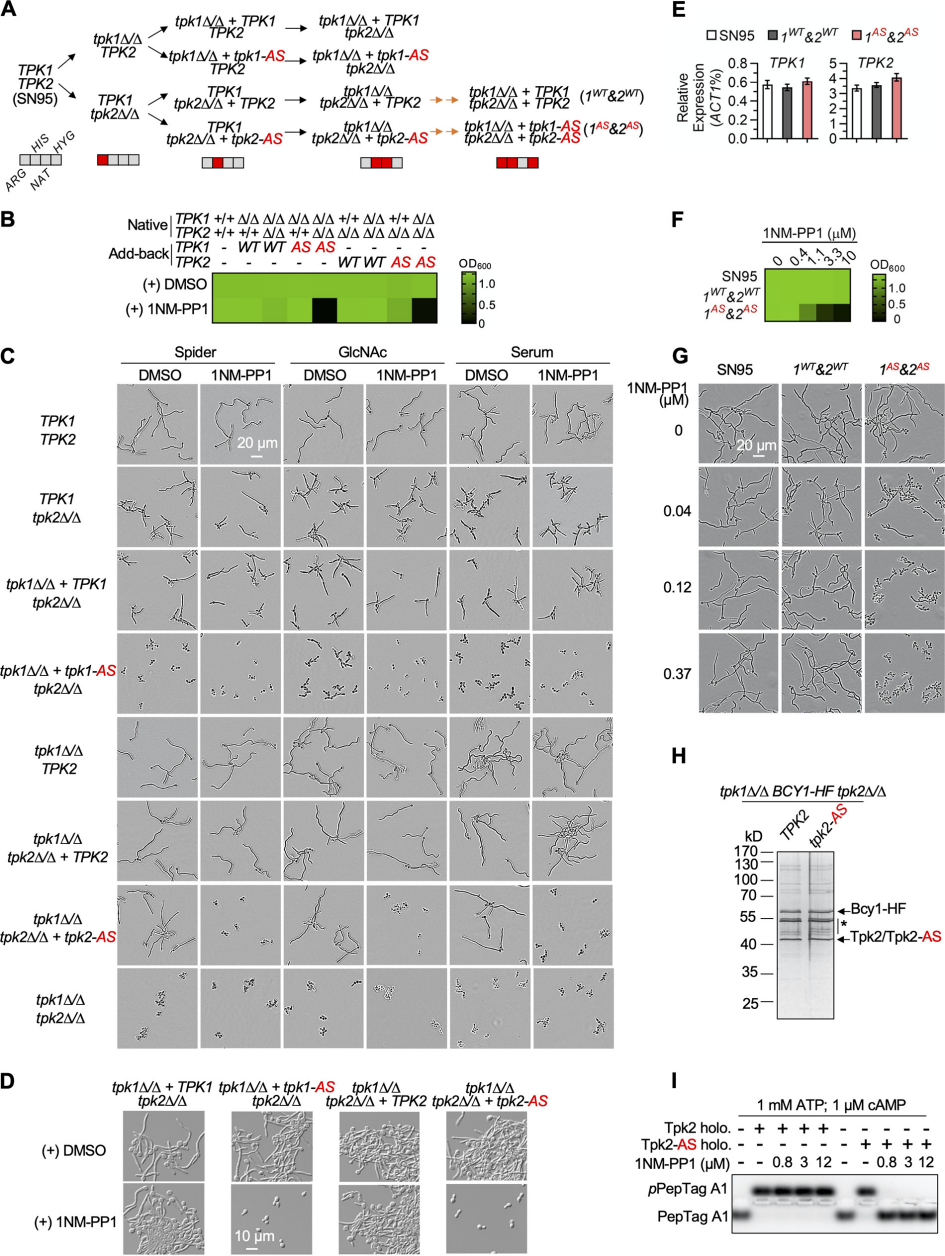
Inhibition of AS Tpk1 and Tpk2 by 1NM-PP1 blocks C. albicans growth and hyphal morphogenesis. (A) A flowchart outlining the strain construction strategy used to make the *TPK1* and *TPK2* AS mutants. A black arrow indicates deletion or complementation at both alleles using a transient CRISPR strategy (23). Two orange arrows indicate the sequential complementation of both alleles using homologous recombination. The marker code utilizes gray to indicate auxotrophy for the indicated amino acid or sensitivity to the indicated drug. Red indicates prototrophy for the amino acid or resistance to the drug indicated. (B) Treatment with 1NM-PP1 inhibits the growth of AS *TPK1* or *TPK2* strains. C. albicans strains were grown overnight to saturation in YPD and then diluted to an optical density at 600 nm (OD_600_) of 0.002 into fresh YPD containing either 2.5 μM 1NM-PP1 or dimethyl sulfoxide (DMSO) vehicle control. Strains were grown at 30°C for 24 h and growth was assessed by OD_600_ (see color bar). (C) Treatment with 2.5 μM 1NM-PP1 inhibits filamentation of the AS *TPK2* strain. Strains were grown at 37°C in either YPD with 10% serum; Spider medium; or yeast nitrogen base supplemented with 11 mM glucose, 2% casamino acid, and 5 mM GlcNAc (YNBNAG) for 4 h. (D) Treatment with 5 μM 1NM-PP1 inhibits filamentation of the AS *TPK1* and *TPK2* strains in RPMI with 10% serum and 5% CO_2_ at 37°C. Strains were grown for 4 h. (E) *TPK1* expression and *TPK2* expression are not significantly different in wild-type or AS C. albicans strains. Transcript levels were normalized to *ACT1*. Data are presented as mean ± SD of technical triplicates. (F) A strain of C. albicans with both AS *TPK1* and *TPK2* alleles exhibited analog-specific inhibition of growth. C. albicans strains were grown as described in B. (G) Treatment with 1NM-PP1 inhibits filamentation in a strain of C. albicans with both AS *TPK1* and *TPK2* alleles. Strains were grown in YNBNAG at 37°C for 4 h. (H) Silver staining of the purified Tpk2/Bcy1 holoenzyme. Strains were grown to log phase in YPD, and then the PKA holoenzyme was purified using a flag-affinity purification as described previously (24). HF represents His6-Flag3. The purified product was run on a 10% SDS-PAGE gel and silver stained. *, impurities. (I) PKA kinase assays were performed using the Peptag kit (Promega). The nonphosphorylated peptide contains one positive charge (+1) and migrates toward the cathode. The phosphorylated peptide contains one negative charge (−1) and migrates toward the anode. Products were resolved on a 0.8% agarose gel in 50 mM Tris-HCl (pH 8.0). The gel was imaged with an Alexa 568 channel. In all panels, the acronym AS stands for analog sensitive. All experiments were performed in biological duplicate.

Here, we first describe the construction and characterization of analog-sensitive (AS) versions of the catalytic subunits of PKA in C. albicans. To do so, we constructed vectors containing wild-type (WT) *TPK1* or *TPK2* alleles. M170G or M180G substitutions were then introduced into the *TPK1* or *TPK2* constructs, respectively, based on the fact that equivalent mutations in Saccharomyces cerevisiae Tpk proteins confer 1NM-PP1 sensitivity (17). The WT and AS alleles were then used to complement the corresponding homozygous deletion mutant at the native loci. Native *TPK2* (or *TPK1*) was then deleted to ensure that the reintroduced WT or AS *TPK1* (or *TPK2*) alleles were the sole source of PKA activity in each strain (Fig. 1A). The C. albicans *tpk1 tpk2* homozygous deletion mutant exhibits a significant growth defect, despite being viable (18). To functionally characterize our newly constructed strains, we monitored growth in the absence and presence of 1NM-PP1. In the absence of 1NM-PP1, WT or AS alleles of *TPK1* or *TPK2* supported normal growth in rich medium (yeast extract-peptone-dextrose [YPD]) (Fig. 1B). The addition of 1NM-PP1 inhibited the growth of strains expressing either *TPK1* or *TPK2* AS alleles as the only catalytic subunit of PKA (“*tpk1Δ/Δ*+*tpk1-AS tpk2Δ/Δ*” or “*tpk1Δ/Δ tpk2Δ/Δ*+*tpk2-AS*”). The growth of the strains expressing only WT alleles of *TPK1* or *TPK2* (“*tpk1Δ/Δ*+*TPK1 tpk2Δ/Δ*” or “*tpk1Δ/Δ tpk2Δ/Δ*+*TPK2*”) was not affected by 1NM-PP1 (Fig. 1B).

In C. albicans, the yeast-to-hyphal transition is largely dependent on PKA signaling. We assessed the ability of the PKA AS strains to filament following 3 to 4 hours of exposure to 1NM-PP1 under the filamentation-inducing conditions of Spider medium, GlcNAc, or serum at 37°C. Under all these conditions, a *tpk1 tpk2* homozygous deletion mutant was blocked in hyphal morphogenesis (Fig. 1C). Similarly, treatment with 1NM-PP1 blocked filamentation in the *tpk1Δ/Δ tpk2Δ/Δ*+*tpk2-AS* strain; as expected, the *tpk1Δ/Δ tpk2Δ/Δ*+*TPK2* strain remained filamentous in the presence of analog (Fig. 1C). Unlike *TPK2*, *TPK1* alone is unable to support wild-type levels of filamentation, as strains expressing WT *TPK1* as the sole source of the PKA catalytic subunits formed shorter filaments than the WT control (Fig. 1C). Interestingly, cells remained in yeast form in the *tpk1Δ/Δ*+*tpk1-AS tpk2Δ/Δ* strain even in the absence of 1NM-PP1, suggesting the allele was not fully functional. When we examined a more host-relevant condition that strongly induces filamentation (RPMI with 10% serum in 5% CO_2_ at 37°C), the *tpk1-AS* mutant could support C. albicans hyphal morphogenesis in the absence of 1NM-PP1 to a similar level as the WT *TPK1* allele (Fig. 1D). However, filamentation of the *tpk1-AS* strain was blocked in these culture conditions with 1NM-PP1. Of note, all 1NM-PP1-dependent blocks in filamentation could be observed after 1 h of exposure (data not shown), highlighting the utility of these AS mutants for studying the dynamic process of morphogenesis.

Next, to generate a strain of C. albicans where both *TPK1* and *TPK2* are replaced with AS alleles, we reintroduced two copies of the AS *TPK1* allele into the *tpk1Δ/Δ tpk2Δ/Δ*+*tpk2-AS* strain at the native loci (Fig. 1A, “1^AS^&2^AS^”). We also generated a strain reconstituted with all four WT *TPK* alleles (Fig. 1A, “1^WT^&2^WT^”) as a control. Both strains expressed *TPK1* and *TPK2* at levels comparable to the wild-type parental strain (Fig. 1E). The strain carrying both AS *TPK1* and *TPK2* alleles exhibited dose-dependent inhibition of growth and filamentation in the presence of 1NM-PP1 (Fig. 1F and G).

Lastly, to provide biochemical evidence for the inhibition of the AS Tpk2 by 1NM-PP1, we purified the Tpk2/Bcy1 holoenzyme from the *tpk1Δ/Δ tpk2Δ/Δ*+*TPK2* and *tpk1Δ/Δ tpk2Δ/Δ*+*tpk2-AS* strains that were modified to express the C-terminal His_6_-FLAG_3_-tagged Bcy1. The products after one-step Flag-affinity purification were resolved on an SDS-PAGE gel, and silver staining was used to confirm the presence of both components of the holoenzyme (Fig. 1H). Using an *in vitro* PepTag PKA activity assay (Promega), we confirmed that the Tpk2/Bcy1 holoenzymes phosphorylate a fluorescent peptide substrate in the presence of cAMP. This activity was sensitive to inhibition by 1NM-PP1 in the holoenzyme purified with the AS Tpk2 but not the wild-type version of the kinase (Fig. 1I).

**FIG 2:**
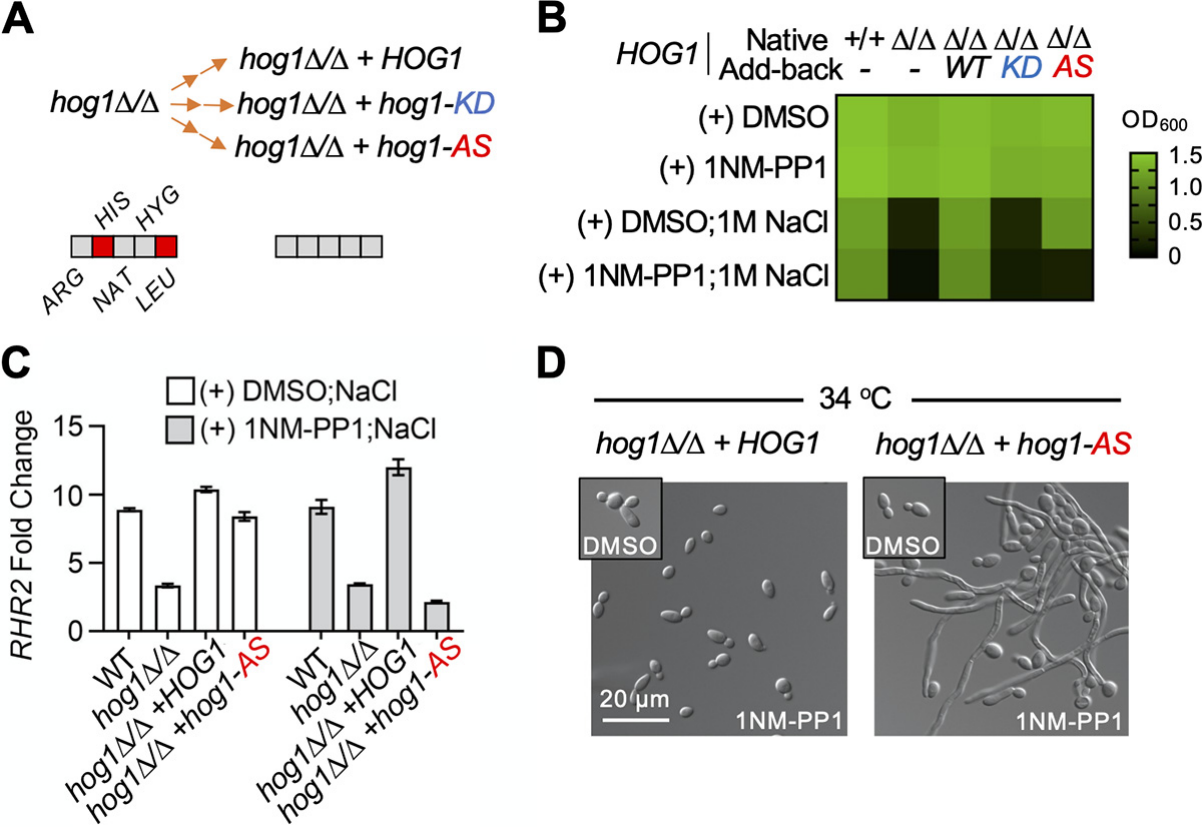
Inhibition of AS Hog1 by 1NM-PP1 results in hypersensitivity to osmotic stress and hyperfilamentation. (A) A flowchart outlining the strain construction strategy used to make the *HOG1* mutants. The orange arrow indicates the sequential complementation of both alleles using conventional homologous recombination with a recyclable cloNAT-resistant cassette. The marker availability is coded as described in Fig. 1A. (B) A growth defect was observed in the AS *HOG1* strain treated with 1 M NaCl and 1NM-PP1. C. albicans strains were grown overnight to saturation in YPD and then diluted to an OD_600_ of 0.002 into fresh YPD in the absence or presence of 2.5 μM 1NM-PP1 or 1 M NaCl, as indicated. Strains were grown at 30°C for 24 h, and growth was assessed by OD_600_ (see color bar). (C) *RHR2* transcript levels are elevated in response to osmotic stress. The AS allele of *HOG1* rescued the increased expression in *RHR2* transcript levels only in the absence of 1NP-PP1. The indicated strains grown in YPD were treated with NaCl (0.8 M for 15 min) after 15-min exposure to DMSO or 1 μM 1NM-PP1. Transcript levels were normalized to *ACT1* and are reported as fold change relative to the absence of NaCl. Data are presented as mean ± SD of technical triplicates. (D) 1NM-PP1 treatment induces filamentation of the AS *HOG1* strain. Strains were grown at 34°C for 8 h. The inset depicts the morphology of cultures in the absence of 1NM-PP1. In all panels, the acronym AS stands for analog sensitive. All experiments were performed in biological duplicate.

Next, we generated and characterized a Hog1 AS mutant in C. albicans. To do so, we constructed a *SAT1*-flipper vector (19) containing a wild-type *HOG1* allele. Then, point mutations conferring N100A or D144A substitutions were introduced into the *HOG1* sequence to generate either an AS or kinase dead (KD) mutant, respectively. The vectors were introduced into a *hog1Δ/Δ* (20) mutant at the native locus to generate WT, AS, or KD Hog1 strains (Fig. 2A).

Complementation of the *hog1* homozygous deletion mutant with either the WT or AS allele (in the absence of the analog), but not the KD allele, restored C. albicans growth under osmotic stress induced by 1 M NaCl (Fig. 2B). However, the AS allele of *HOG1* in the presence of 1NM-PP1 was unable to support C. albicans growth under this condition (Fig. 2B). Complementation with either the WT or the AS allele also restored the Hog1-mediated transcriptional response to osmotic stress, as measured by the expression of the glycerol 3-phosphatase gene *RHR2* (Fig. 2C). Treatment with 1NM-PP1 inhibited the induction of *RHR2* in the strain of C. albicans with AS Hog1 (Fig. 2C). Finally, previous work identified Hog1 as a repressor of filamentation (9). 1NM-PP1 treatment induced filamentation of the *hog1Δ/Δ*+*hog1-AS* strain at 34°C, which is a temperature nonpermissive for the *hog1Δ/Δ*+*HOG1* strain to filament (Fig. 2D).

The work presented here describes strains that can be employed to facilitate a deeper understanding of how PKA and Hog1 regulate morphogenesis and stress responses in C. albicans. Our characterization suggests that the AS Tpk2 and AS Hog1 kinases have wild-type-like function and are sensitive to specific inhibition by 1NM-PP1. The AS *TPK1* allele, however, is likely hypomorphic, as it supports hyphal morphogenesis only under specific conditions. The partial loss of function is not uncommon for AS kinases (17). Importantly, all strains generated allow for at least one selectable marker to be used for further genetic modification. Future work will continue to develop AS versions of other C. albicans kinases of interest, especially for those proteins explored as potential targets for antifungal development, such as the casein kinases Yck2 and Hrr25 (21, 22).
